# Sodium Channel–Dependent and –Independent Mechanisms Underlying Axonal Afterdepolarization at Mouse Hippocampal Mossy Fibers

**DOI:** 10.1523/ENEURO.0254-18.2018

**Published:** 2018-08-23

**Authors:** Shunsuke Ohura, Haruyuki Kamiya

**Affiliations:** Department of Neurobiology, Hokkaido University Graduate School of Medicine, Sapporo 060-8638, Japan

**Keywords:** Action potential, afterdepolarization, axon, CA3, hippocampus, mossy fiber

## Abstract

Action potentials propagating along axons are often followed by prolonged afterdepolarization (ADP) lasting for several tens of milliseconds. Axonal ADP is thought to be an important factor in modulating the fidelity of spike propagation during repetitive firings. However, the mechanism as well as the functional significance of axonal ADP remain unclear, partly due to inaccessibility to small structures of axon for direct electrophysiological recordings. Here, we examined the ionic and electrical mechanisms underlying axonal ADP using whole-bouton recording from mossy fiber terminals in mice hippocampal slices. ADP following axonal action potentials was strongly enhanced by focal application of veratridine, an inhibitor of Na^+^ channel inactivation. In contrast, tetrodotoxin (TTX) partly suppressed ADP, suggesting that a Na^+^ channel–dependent component is involved in axonal ADP. The remaining TTX-resistant Na^+^ channel–independent component represents slow capacitive discharge reflecting the shape and electrical properties of the axonal membrane. We also addressed the functional impact of axonal ADP on presynaptic function. In paired-pulse stimuli, we found that axonal ADP minimally affected the peak height of subsequent action potentials, although the rising phase of action potentials was slightly slowed, possibly due to steady-state inactivation of Na^+^ channels by prolonged depolarization. Voltage clamp analysis of Ca^2+^ current elicited by action potential waveform commands revealed that axonal ADP assists short-term facilitation of Ca^2+^ entry into the presynaptic terminals. Taken together, these data show that axonal ADP maintains reliable firing during repetitive stimuli and plays important roles in the fine-tuning of short-term plasticity of transmitter release by modulating Ca^2+^ entry into presynaptic terminals.

## Visual Abstract

**Figure:**
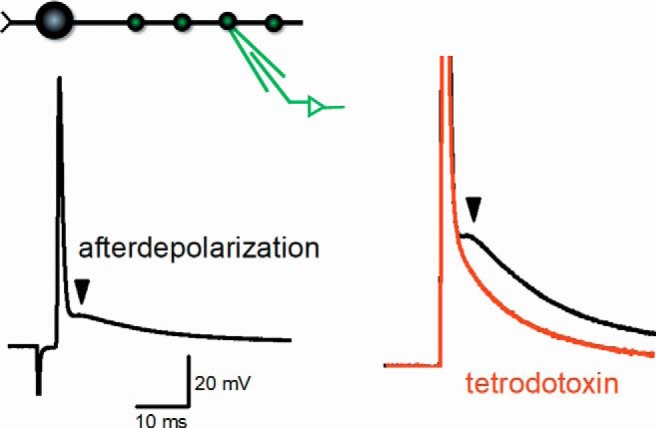

## Significance Statement

Axonal action potentials are often followed by depolarizing or hyperpolarizing afterpotentials. This study illuminated the mechanisms of ADP in the hippocampal mossy fibers, where morphologic as well as biophysical data were accumulated. We found that slow activating Na^+^ channels are partly involved in ADP. Capacitive components also substantially contribute to ADP, suggesting that axonal shape and electrical properties are optimized for high-fidelity propagation during repetitive stimuli. We also tested the roles of ADP in the activity-dependent tuning of the presynaptic Ca^2+^ current. Action potential–driven Ca^2+^ entry into the axon terminals was facilitated by paired stimuli, possibly due to Ca^2+^ current facilitation by ADP. Therefore, ADP contributes to fine-tuning of transmitter release and ensures high-fidelity spiking of axons.

## Introduction

The propagation of action potentials along axons is a fundamental process in the nervous system to reliably carry neuronal information to the target cells (Debanne et al., 2011; Kole and Stuart, 2012). Mechanisms enabling ultrafast and reliable spike signaling were studied extensively in various preparations of the central nervous system, including myelinated and unmyelinated axons. However, the mechanisms and functional consequences of axonal ADP, which often follows axonal action potentials and lasts for several tens of milliseconds, remain to be elucidated. Thus far, few studies have directly addressed the mechanisms for the generation of axonal ADP in the central nervous system, except for the calyx of Held axon terminals (Borst et al., 1995; Kim et al., 2010), which enable direct electrophysiological recording from the large axon terminals.

As local application of the Na^+^ channel blocker tetrodotoxin (TTX) minimally affected ADP recorded from the calyx of Held axon terminals, the slow capacitive discharge of axonal membrane has been implicated in the generation of axonal ADP (Borst et al., 1995), as suggested in a previous study on lizard myelinated motor axons using intra-axonal recordings (Barrett and Barrett, 1982).

In this context, it should be noted that ADP has been demonstrated to exhibit clear dependence on the initial membrane potentials. On depolarization of the axonal membrane, the amplitude of ADP decreased and occasionally reversed in polarity (Begum et al., 2016; Sierksma and Borst, 2017). This indicated that ADP not only consists of passive component, but also incorporates some active component due to the activation of voltage-dependent conductance.

On the other hand, ADP was found to be largely suppressed by the application of low-concentration TTX to the same calyx of Held axon terminals (Kim et al., 2010). In that report, the author raised the possibility that certain subtypes of voltage-dependent Na^+^ channels mediate ADP. Na^+^ channels are functionally classified into at least three distinct subtypes: the transient-type (I_NaT_), persistent-type (I_NaP_), and resurgent-type (I_NaR_). Involvement of slowly activating resurgent Na^+^ current (I_NaR_) in axonal ADP was suggested in the study by Kim et al. (2010) because dialysis of a small peptide fragment of the β4 subunit of Na^+^ channels, which is an essential molecular component for I_NaR_, selectively enhanced ADP. As the reason for the controversial conclusion of these studies (Borst et al., 1995; Kim et al., 2010) remains unclear, thorough investigation is needed to clarify the mechanisms underlying axonal ADP in the central nervous system.

The functional significance of axonal ADP must also be addressed. It was widely considered that ADP lowers the threshold of subsequent action potentials, thereby enhancing the fidelity of spiking during high-frequency neuronal activity (Barrett and Barrett, 1982). Moreover, the prolonged time course of ADP was suggested to improve fine-tuning of presynaptic functions, such as action potential–driven Ca^2+^ entry and subsequent transmitter release, by affecting the voltage-dependent conductance shaping axonal action potentials such as Na^+^ and K^+^ channels. However, detailed analysis of the axon terminals of calyx of Held revealed that ADP minimally affects Ca^2+^ currents by balancing and cancelling out changes in the driving force and gating of voltage-dependent Ca^2+^ channels (Clarke et al., 2016).

In this study, we examined the mechanisms underlying axonal ADP at hippocampal mossy fibers, where it was reported that prominent ADP follows axonal action potentials (Geiger and Jonas, 2000). Whole-cell recording from large axon terminals of hippocampal mossy fibers in combination with numerical simulation based on a realistic model of hippocampal mossy fibers (Engel and Jonas, 2005) was adopted to examine the ionic and electrical mechanisms underlying axonal ADP. We also closely investigated the influence of axonal ADP on action potential waveforms and Ca^2+^ current in presynaptic terminals to evaluate its functions for the activity-dependent tuning of presynaptic Ca^2+^ entry and subsequent transmitter release.

## Materials and Methods

### Animals and slice preparations

C57BL/6J mice of either sex were used in this study and were treated according to the guidelines for the care and use of laboratory animals of Hokkaido University. Transverse hippocampal slices of 300-µm thickness were prepared from p14-29 mice (number of animals = 46) as described previously (Kamiya, 2012; Ohura and Kamiya, 2018). Animals were anesthetized with ether, and the brain was dissected in an ice-cold sucrose solution containing the following: 40 mm NaCl, 25 mm NaHCO_3_, 10 mm glucose, 150 mm sucrose, 4 mm KCl, 1.25 mm NaH_2_PO_4_, 0.5 mm CaCl_2_, and 7 mm MgCl_2_. Transverse slices were cut using a VT1200S microslicer (Leica Biosystems). The slices were then exchanged in a NMDG-HEPES recovery solution containing the following: 93 mm NMDG, 2.5 mm KCl, 1.2 mm NaH_2_PO_4_, 30 mm NaHCO_3_, 20 mm HEPES, 25 mm glucose, 5 mm Na-ascorbate, 2 mm thiourea, 3 mm Na-pyruvate, 10 mm MgSO_4_, and 0.5 mm CaCl_2_ and incubated for no longer than 15 min at 30–32°C (Ting et al., 2014). Then the solution was exchanged with standard artificial cerebrospinal fluid (ACSF) containing the following: 127 mm NaCl, 1.5 mm KCl, 1.2 mm KH_2_PO_4_, 26 mm NaHCO_3_, 10 mm glucose, 2.4 mm CaCl_2_, and 1.3 mm MgSO_4_, and the slices were kept in an interface-type chamber saturated with 95% O_2_ and 5% CO_2._ The slices were incubated in the ACSF at room temperature for at least 1 h before experiments.

### Electrophysiology

Mossy fiber boutons (MFBs) were visually identified under a microscope with IR-DIC optics (BX-51 WI, Olympus), as reported previously (Ohura and Kamiya, 2018; see also Geiger and Jonas, 2000; Alle and Geiger, 2006). Slices were continuously perfused at ∼2 ml/min with ACSF. In addition, the slice surface of the recording site was locally perfused with the drug-containing solution at ∼0.2 ml/min though a flow pipe with a 250-µm open-tip diameter connected to an electromagnetic valve system (Valve Bank, Automate Scientific). All recordings were made at room temperature (25 ± 1°C) with a patch clamp amplifier (MultiClamp700B, Molecular Devices). Patch pipettes (typically 8–14 MΩ electrode resistance) were made from borosilicate glass with a microelectrode puller (Sutter P-97, Sutter Instruments). Ca^2+^-free ACSF (an equal concentration of Mg^2+^ replaced Ca^2+^; 0 mm CaCl_2_ and 3.7 mm MgSO_4_) was perfused in the bath and focally applied to minimize the synaptic input from the surrounding cells. In whole-cell current clamp recordings, the patch pipettes were filled with an intracellular solution containing the following: 140 mm K-gluconate, 10 mm KCl, 0.2 mm EGTA, 2 mm MgATP, and 10 mm HEPES, adjusted to pH 7.2. Electrical stimuli for 200 µs were given every 10 s at the granule cell layer of the dentate gyrus, except for in experiments shown in Fig. 3*E–I*, in which stimuli were delivered every 30 s. The liquid junction potential was estimated as –15 mV using pCLAMP10 software and compensated for the holding potential. The series resistance and electrode capacitance were compensated before measurement. The recordings were adopted only when the resting membrane potentials were between –60 and –85 mV immediately after the break-in. The membrane potential was set to –80 mV manually by applying a small holding current if necessary (Geiger and Jonas, 2000). Input resistance was continuously monitored by injecting a hyperpolarizing current pulse (–10 pA, 300 ms) in each sweep. MFBs with input resistance larger than 800 MΩ and series resistance lower than 70 MΩ were used for later analyses. The data were excluded if the series resistance changed by >20% of the initial value during the recording. In voltage-clamp experiments, pipettes were filled with an internal solution containing the following: 145 mm CsCl, 2 mm MgCl_2_, 2 mm Na_2_ATP, 0.3 mm NaGTP, 5 mm Na_2_-phosphocreatinine, 10 mm HEPES, and 10 mm EGTA, adjusted to pH 7.2. To record the Ca^2+^ current, ACSF containing 1 µm tetrodotoxin (TTX), 20 mm tetraethylammonium chloride (TEA), and 5 mm 4-aminopyridine (4AP) was focally applied to the recording sites. To adjust the osmolarity of the solution, the concentration of NaCl in the ACSF was lowered by 20 mm. Leakage and capacitive currents were subtracted online using P/4 procedures. Series resistance (57.2 ± 3.3 MΩ, *n* = 7) was compensated by 50%–70%. In some recordings, unclamped tail currents with a very slow time course appeared in an all-or-none manner, possibly reflecting spiking of the neighboring axons or boutons. These recordings with a time constant longer than 400 µs were excluded from analysis. Signals were filtered at 10 kHz with a 4-pole Bessel filter and were digitized at 20 kHz with a Digidata 1322A interface and 16-bit resolution (Molecular Devices). All data were acquired and analyzed offline with pClamp 10.7 software (Molecular Devices) and Origin 8J or 2015 (OriginLab).

### Simulation

The simulated membrane potential (V_m_) at the hippocampal mossy fibers was calculated according to the model suggested by Engel and Jonas (2005) based on the data recorded from mossy fiber boutons. The model basically assumed a Hodgkin–Huxley-type model (Hodgkin and Huxley, 1952) adapted to channels in mossy fiber terminals, and K^+^ channel inactivation was implemented multiplicatively with parameters of recombinant K_V_1.4 channels (Wissmann et al., 2003). Simulations were performed using NEURON 7.5 for Windows (Hines and Carnevale, 1997). The passive electrical properties of the axon were assumed to be uniform, with a specific membrane capacitance Cm of 1 µF cm^−2,^ a specific membrane resistance Rm of 10,000 Ω cm^2^, and an intracellular resistivity Ri of 110 Ω cm. The structure of the mossy fiber was approximated by a soma (diameter, 10 µm), 10 axonal cylinders (diameter, 0.2 µm; length, 100 µm), and 10 *en passant* boutons (diameter, 4 µm). The number of segments was 1 µm^−1^, and the time step was 5 µs in all simulations. The resting potential was assumed to be −80 mV. The reversal potential of the leak conductance was set to −81 mV to maintain stability. Voltage-gated Na^+^ channels, K^+^ channels, and leakage channels were inserted into the soma, axon, and boutons, respectively. The Na^+^ conductance density was set to 50 mS cm^−2^ for the axon and boutons and 10 mS cm^−2^ for the soma. The K^+^ conductance density was set to 36 mS cm^−2^ throughout all parts of the neurons. Action potentials were evoked by injection of depolarizing current into the 9th bouton (1 ms, 0.15 nA) or the soma (2 ms, 0.2 nA) in the simulation shown in Figs. 4*G*and 4*H*, respectively. We conducted stimulation on the last (10th) bouton in a “pearl chain structure” to avoid sealed end effects. The equilibrium potentials for Na^+^ and K^+^ ions were assumed to be +50 mV and −85 mV, respectively.

### Statistics

All data are expressed as the mean ± SEM. Statistical analysis was performed by nonparametric two-sided tests (Wilcoxon signed-rank test for paired data and Mann–Whitney *U* test for unpaired data), and a *p*-value of <0.05 was accepted for statistical significance. All statistical analyses were conducted using R software (version 3.4.1).

### Chemicals

Veratridine was purchased from Sigma-Aldrich. Tetrodotoxin was purchased from Funakoshi. All other chemicals were purchased from Wako Pure Chemical.

## Results

### Slow ADP following axonal action potentials at hippocampal mossy fibers

**Figure 1.**
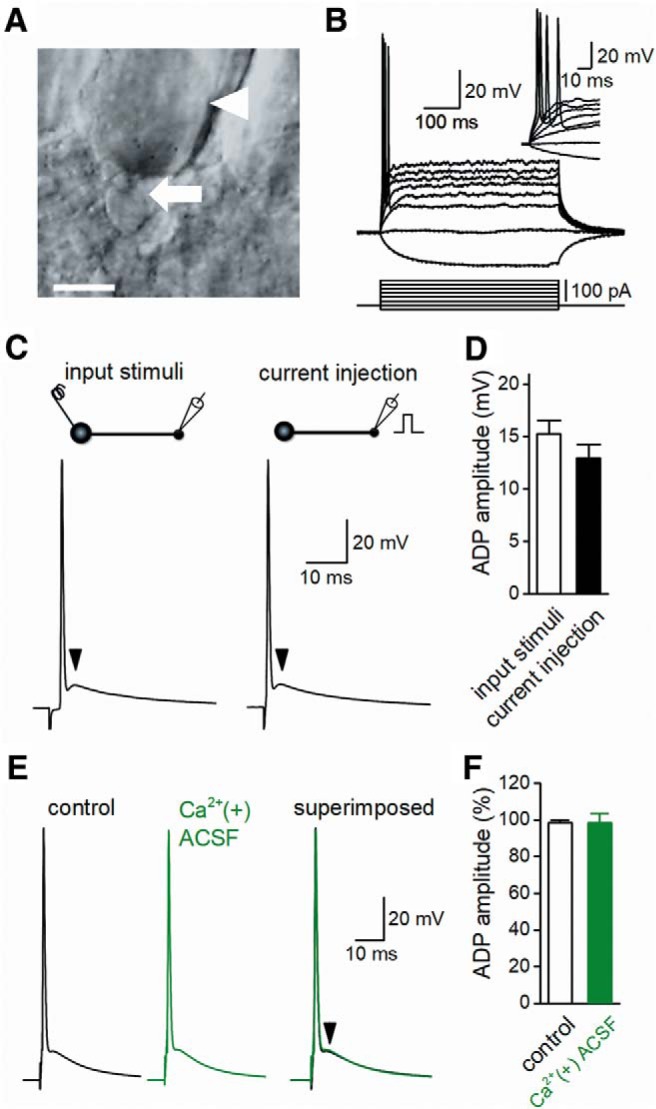
Prominent ADP following action potentials at MFBs in mouse hippocampal slices. ***A***, IR-DIC image of the recorded bouton (arrow) and CA3 pyramidal cell (arrowhead). Scale bar represents 5 µm. ***B***, Whole-cell recordings confirmed typical membrane potential responses of MFBs with single action potentials in response to the current injection (–20- to 120-pA pulses for 500 ms, 20 pA each step). ***C***, Action potential of MFBs evoked by input stimuli at the granule cell layer (left) and current injection at the recorded boutons (right). The arrowheads represent the peaks of ADP. ***D***, Summary data of the amplitude of ADP elicited by input stimuli (open column; *n* = 20) or by current injection (closed column, *n* = 6). ***E***, Effects of Ca^2+^-containing ACSF on action potentials elicited by current injection. ***F***, Summary of ADP amplitude in the absence (open column) and presence (closed column) of Ca^2+^ (*n* = 8).

To characterize the detailed properties of ADP following action potentials, whole-cell current clamp recordings were made from large MFBs in mouse hippocampal slices (Fig. 1*A*). Long depolarizing current pulses (20–120 pA, 500 ms) in the current clamp configuration constantly evoked single action potentials to increasing currents (Fig. 1*B*), as reported previously (Geiger and Jonas, 2000; Bischofberger et al., 2006). Input resistance and the time constant of MFBs were also measured from voltage responses to hyperpolarizing current injection of –10 pA for 300 ms. Input resistance and the decay time constant were 1.06 ± 0.06 GΩ and 36.4 ± 3.3 ms, respectively (*n* = 20), and were consistent with those reported in previous studies (Bischofberger et al., 2006; Szabadics and Soltesz, 2009). Action potentials were also evoked by extracellular stimulation at the granule cell layer of the dentate gyrus (Fig. 1*C*, left). The amplitude and half-width of action potentials evoked by stimulation of granule cells were 114.5 ± 2.4 mV and 863 ± 34 µs, respectively (*n* = 20). Alternatively, injection of short current pulses (500–800 pA for 1 ms) to the recorded bouton also elicited action potentials (Fig. 1*C*, right). The amplitude and half-width of the action potentials evoked by current injection were 113.8 ± 2.6 mV and 918 ± 26 µs, respectively (*n* = 6), which were not significantly different from those evoked by stimulation at the granule cell layer (*p* = 0.700 and *p* = 0.422 for amplitude and half duration, respectively).

In each stimulation mode of input stimulation or current injection, a small and prolonged ADP followed the action potentials in MFBs (Geiger and Jonas, 2000; Kamiya et al., 2002), as shown in Fig. 1*C* (arrowheads). The amplitude and decay time constant of ADP by stimulation of input fibers were 15.3 ± 1.3 mV and 41.6 ± 3.8 ms, respectively (*n* = 20), which were not significantly different from those evoked by current injection to the recorded boutons (13.0 ± 1.3 mV and 43.6 ± 5.5 ms), as shown in Fig. 1*D* (*p* = 0.494 for amplitude and *p* = 0.700 for decay time constant). As these data were recorded in Ca^2+^-free ACSF to minimize the influence of synaptically released transmitters (Ohura and Kamiya, 2018), ADP was considered to have originated intrinsically from the membrane properties of MFBs.

Previous reports suggested that R-type Ca^2+^ channels contribute to ADP in CA1 pyramidal neurons (Metz et al., 2005), and mossy fiber terminals contain R-type Ca^2+^ channels (Gasparini et al., 2001; Li et al., 2007). However, any possible contribution of R-type Ca^2+^ channels to ADP generation was omitted in the recording in Ca^2+^-free solution. To test for possible contribution of R-type Ca^2+^ channels in axonal ADP in the mossy fibers, we examined the effects of Ca^2+^-containing ACSF and found no significant changes in action potentials or ADP generated by short current injection (*p* = 0.742 and *p* = 0.742, respectively, *n* = 8, Fig. 1*E, F*). This suggests that ADP at mossy fiber terminals was not mediated by Ca^2+^ channel–dependent components.

### Voltage dependence of axonal ADP

**Figure 2.**
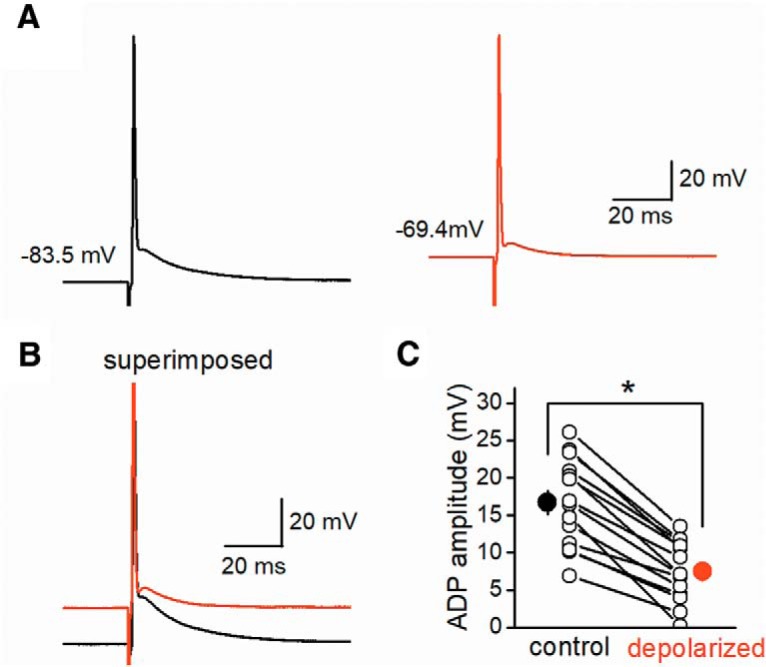
Dependence of ADP on initial membrane potentials. ***A***, Action potentials were recorded at control (black) and depolarized (red) membrane potentials by injecting constant currents into the recorded boutons. Although the peak of action potentials was unaffected by changes in initial membrane potentials, the amplitude and the time course of ADP were significantly altered by changes in the membrane potentials. ***B***, Superimposed traces illustrate the voltage-dependence of ADP. ***C***, Summary data for ADP amplitude recorded at control and depolarized initial membrane potentials (*n* = 14, *p < 0.05).

To investigate the mechanisms underlying ADP, we first examined the voltage-dependence of ADP amplitude. For this purpose, membrane potentials were changed by injecting continuous currents into the recording MFBs. At positive membrane potentials relative to resting membrane potentials, the amplitude of ADP was decreased and the waveform was markedly altered (Fig. 2*A, B*). In these experiments, we measured membrane potentials at 5 ms after the peak of the action potentials because the peak of ADP was evident at that time point. The ADP amplitude was 16.7 ± 1.6 mV in the control conditions at –79.1 ± 0.6 mV, and decreased to 7.5 ± 1.1 mV at the depolarized conditions of –66.2 ± 0.9 mV (*n* = 14, *p* = 0.00012, Fig. 2*C*). The negative correlation of ADP with initial membrane potentials was also reported at presynaptic terminals of the calyx of Held (Sierksma and Borst, 2017) or cerebellar basket cells (Begum et al., 2016).

### Sodium channel–dependent component of axonal ADP

**Figure 3.**
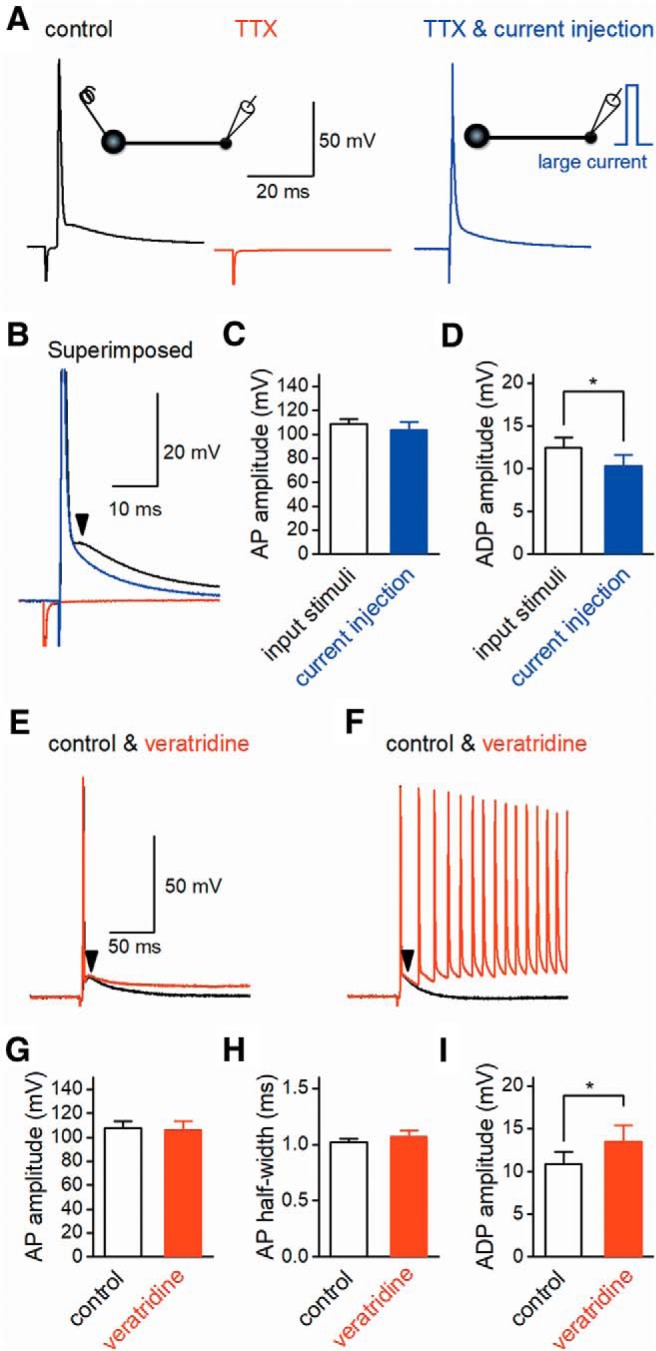
Involvement of Na^+^ channels in axonal ADP. ***A***, Action potentials evoked by input stimuli (black) were completely suppressed by application of 0.5 µm TTX (red). Large current injection into the recorded MFB elicited mock action potentials with similar time courses of action potentials and ADP (blue). ***B***, Superimposed control (black) and mock action potentials (blue) demonstrated that TTX-sensitive components overtook the TTX-resistant slow ADP, suggesting that TTX-sensitive Na^+^-channels partly enhance ADP. ***C***, ***D***, Comparison of control and mock action potentials (**C**) and ADP (***D***) amplitude (*n* = 6, *, *p* < 0.05). ***E***, ***F***, Effects of focal application of 1 µm veratridine, an inhibitor of Na^+^ channel inactivation, on ADP. Veratridine enhanced and prolonged ADP (***E***). In some cases, multiple action potentials (***F***) were overlaid, as shown in the right panel. ***G****–****I***, Summary data for the effects of veratridine on the amplitude (***G***), half-width (***H***) of action potentials, and the amplitude of ADP (***I***; *n* = 6, *, *p* < 0.05).

It was reported that the slow reactivation of resurgent-type Na^+^ currents mediates ADP at the presynaptic terminals of the calyx of Held (Kim et al., 2010). Focal application of 0.5 µM TTX to the recording site completely abolished both action potentials (from 108.9 ± 4.0 to 1.0 ± 0.4 mV, *n* = 6, *p* = 0.0313) and ADP (from 12.4 ± 1.2 to 0.4 ± 0.2 mV, *n* = 6, *p* = 0.0313), as shown in Fig. 3*A*. To test for possible contribution of Na^+^ channels to ADP, we examined the effects of TTX on ADP. For this purpose, brief current pulses of 2000–4300 pA for 1 ms were injected to mimic the short depolarization of action potentials (103.7 ± 6.7 mV, *n* = 6) in the presence of TTX (Fig. 3*A*). The peak amplitude of ADP was decreased to 10.3 ± 1.3 mV by TTX (*n* = 6), and the difference in ADP amplitudes between with and without TTX was significant (*p* = 0.0313), as shown in Fig. 3*D*. This suggests that TTX-sensitive Na^+^-channels partly mediate ADP at MFBs.

On the other hand, a previous study revealed that veratridine, an inhibitor of sodium channel inactivation, markedly enhanced and prolonged ADP at the calyx of Held presynaptic terminals (Kim et al., 2010). Therefore, we examined the effects of veratridine on action potentials and ADP at hippocampal MFBs. Application of 1 µM veratridine had minimal effects on the action potentials (Fig. 3*E, F*). The amplitude and half-width of action potentials were 107.6 ± 6.0 mV and 1.02 ± 0.03 ms, respectively, in the control conditions, whereas they were 106.0 ± 7.4 mV and 1.07 ± 0.06 ms, respectively, after application of veratridine (*n* = 6). The differences were not significant (*p* = 0.563 and 0.688, Fig. 3*G, H*). However, the amplitude of ADP was significantly increased from 10.8 ± 1.5 to 13.5 ± 1.9 mV by veratridine (*n* = 6, *p* = 0.0313, Fig. 3*I*). During application of veratridine for 10 min, 3 of 6 recordings exhibited mild enhancement and prolongation of ADP, as shown in Fig. 3*E*, but the other 3 recordings accompanied multiple spikes overlaying enhanced ADP (Fig. 3*F*). These results suggest that veratridine-sensitive Na^+^ channels contributed to the generation of ADP, although the amount varied among boutons. As ADP largely remained with TTX (Fig. 3*B, D*), a mechanism other than Na^+^ channels is involved in the generation of ADP.

### Capacitive component of axonal ADP

**Figure 4.**
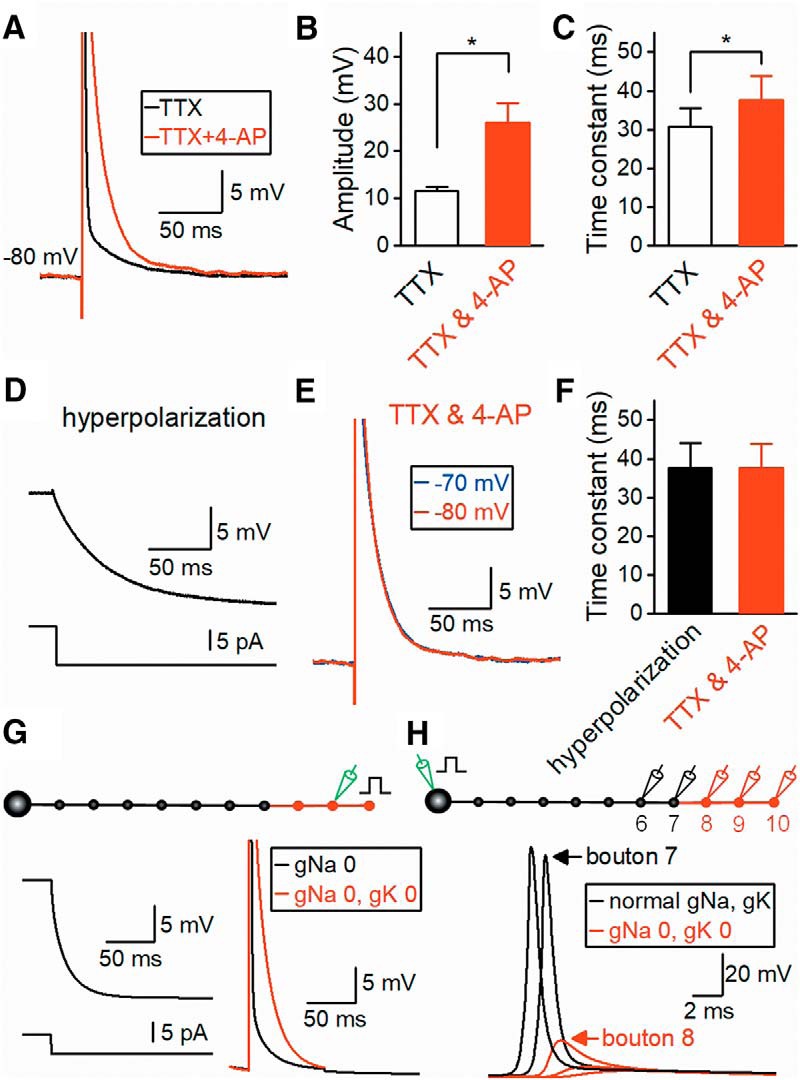
Contribution of capacitive slow discharge in the axonal ADP. ***A***, Mock action potentials (black) elicited by large current injection into MFBs in the presence of TTX were prolonged by the addition of 2 mm 4-AP (red), leaving the capacitive components of the axonal membrane. ***B, C***, Summary data for the amplitude (***B***) and decay time constant (***C***) of ADP (*n* = 8, *, *p* < 0.05). ***D***, Time constant of MFBs measured by hyperpolarizing current injection (–10 pA, 300 ms). ***E***, Comparison of decay time course of capacitive components at different initial membrane potentials of –80 and –70 mV. **F**, Summary data for decay time constants of hyperpolarization (***D***) and the capacitive component of ADP at –80 mV (***E***). ***G***, Simulated membrane potentials in the mossy fiber model by Engel and Jonas (2005) in response to hyperpolarizing current injection into distal MFBs (left). Brief large current injection elicited a similar response to those observed in ***A*** (right) when gNa and gK were omitted from the distal axons shown in red. See Methods for details. ***H***, Simulation of passive propagation of upstream action potentials to distal axons where gNa and gK were omitted.

Next, we addressed the contribution of the component derived from slow capacitive discharge of the axonal membrane (Barrett and Barrett, 1982; Borst et al., 1995; David et al., 1995). As shown in Fig. 3*A*, brief current injection into the MFBs elicited prolonged depolarization resistant to the application of TTX. To measure the components of capacitive discharge, we evaluated the effects of 4-AP, which almost completely blocks potassium channels at MFBs (Alle et al., 2011), on TTX-resistant depolarization. Application of 2 mm 4-AP increased the amplitude of the slow depolarization component at 5 ms from 11.6 ± 0.9 to 26.0 ± 4.0 mV (*n* = 8, *p* = 0.00781), as shown in Fig. 4*A*, *B*. The decay time constant was also increased by 4-AP (from 30.9 ± 4.8 to 37.6 ± 6.1 ms, *n* = 8, *p* = 0.00781, Fig. 4*C*). These results suggest that delayed activation of voltage-dependent K^+^ channels curtails the repolarizing phase of the brief depolarization by TTX, and the voltage responses elicited in the presence of TTX and 4-AP most likely represent capacitive components due to axonal membrane discharge. This notion is supported by the findings that the time constant (37.6 ± 6.1 ms) was not significantly different from that observed in hyperpolarization elicited by stepwise negative current injection (37.7 ± 6.7 ms, *n* = 8; *p* = 0.547, Fig. 4*F*). As expected from the passive nature of capacitive discharge, the time course of this component was voltage-independent (Fig. 4*E*). The amplitudes were 26.0 ± 4.0 mV at –80 mV and 25.3 ± 3.9 mV at –70 mV, and the difference was not significant (*p* = 0.945). The decay time constant recorded at –80 mv (37.6 ± 6.1 ms) was not different from that at –70 mV (35.8 ± 6.1 ms, *n* = 8, *p* = 0.813).

To quantitatively evaluate the contribution of the capacitive component, we also conducted numerical simulation using the model described previously (Engel and Jonas, 2005). This was a realistic mossy fiber model incorporating the compartmental cable model mimicking the morphology of mossy fiber axons, as well as a Hodgkin–Huxley-type gating model of voltage-dependent Na^+^ channels that was obtained experimentally and implemented with inactivation of voltage-dependent K^+^ channels. Hyperpolarization evoked by negative current injection was optimally fitted with the sum of two exponentials, reflecting two electrical components of boutons and the connecting axons, and the time constant of the dominant component was 17.0 ms (Fig. 4*G*, left). The decay time constant for short current injection, in which both Na^+^ and K^+^ conductance was reduced to 0, was 15.0 ms (Fig. 4*G*, right).

We also conducted simulation for passive propagation of the action potentials through a thin axonal cable from the nearby MFBs. For this purpose, we reconstituted the propagation of action potentials elicited by injecting stimuli at the soma and calculated the voltage transients at the distal axons and the MFBs (Fig. 4*H*). Simulation demonstrated that the action potential of nearby MFBs (108.2 mV in amplitude) was passively propagated to the next MFBs (17.3 mV in amplitude). The spatial distribution constant of the passive propagation of transient signal, which we defined in this study as the distance that reduced the size of the transient signal to 1/e (37%) without any active conductance, was estimated as 53.6 µm, and this value was similar with that reported for Purkinje cell axons (Zorrilla de San Martin et al., 2017). Therefore, the capacitive component of ADP may reflect the propagation of action potentials at upstream axons and/or MFBs.

In the absence of active conductance, action potentials passively propagate and decay exponentially with distance. However, this spatial distribution of transient voltage changes is strongly filtered by the electrical cables of thin axons and should be different from and shorter than those of steady-state responses indicated by the length constant, which represents the duration for decay of the size of steady-state responses to 1/e.

These results suggest that the decay time course of ADP is mainly determined by the slow capacitive discharge of the axonal membrane following action potentials. Veratridine-sensitive Na^+^ channels and 4AP-sensitive K^+^ channels additionally contribute to ADP to modulate the time course in mouse hippocampal MFBs.

### Broadening of axonal action potentials by ADP

**Figure 5.**
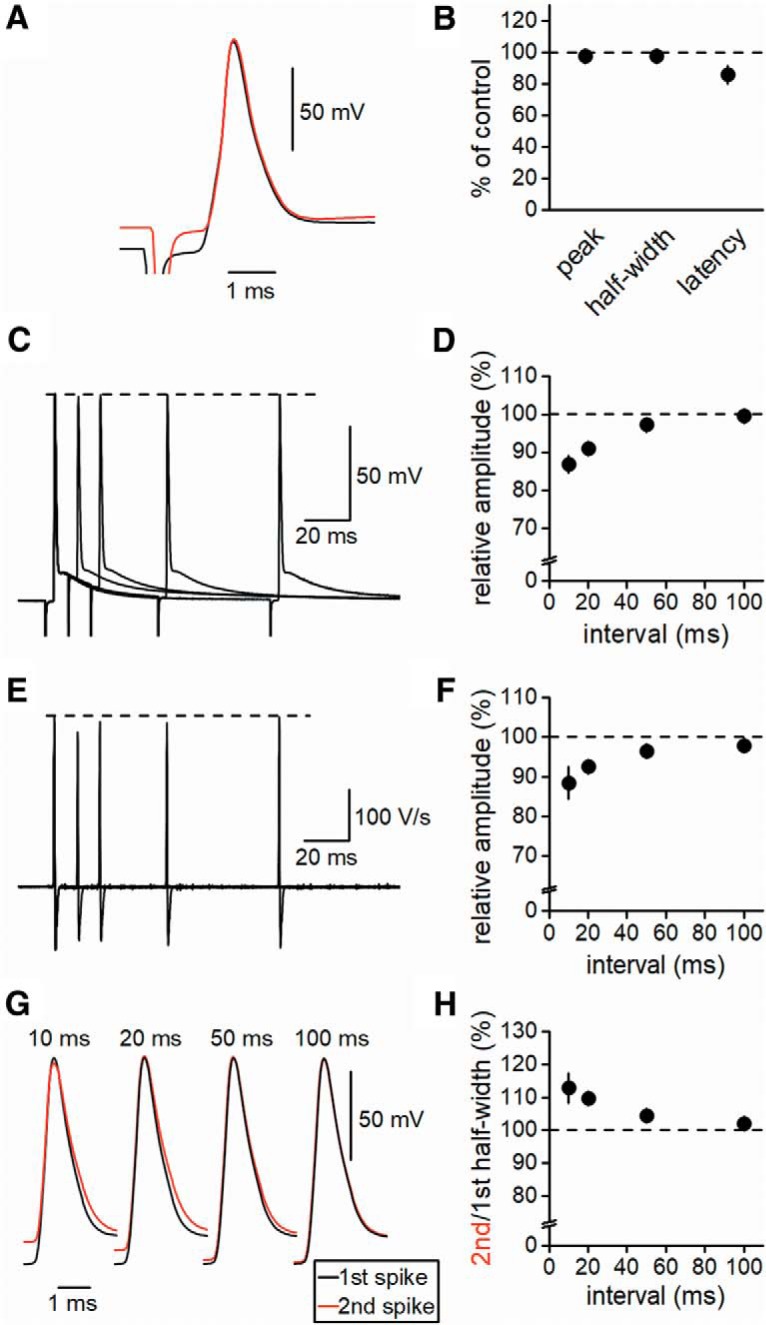
Broadening of axonal action potentials by the preceding ADP. ***A***, Effects of changes in initial membrane potentials on action potentials elicited by stimulation of input fibers recorded at depolarized (red) or resting (black) membrane potentials. ***B***, Summary data for the relative peak, half-width, and latency of action potentials (*n* = 14). ***C***, Superimposed traces of paired-pulse responses at 10-, 20-, 50-, and 100-ms intervals. ***D***, Summary data for the paired-pulse ratio of action potential amplitude measured from the onset of the second action potential (*n* = 8). ***E***, First derivatives calculated from paired-pulse responses are shown in ***C*** and ***F***, Summary data for the paired-pulse ratio of the first derivative waveform (*n* = 8). ***G***, Expanded traces of the first (black) and second (red) action potentials at different interspike intervals. ***H***, Summary data for the paired-pulse ratio of the action potential half-width (*n* = 8).

Our previous study revealed short-term depression of axonal spikes, as recorded by loose-patch-clamp recording (Ohura and Kamiya, 2018). As application of veratridine selectively depressed the second spike with minimal effects on the first spike, we supposed that ADP following the first action potential affects the second action potential by steady-state inactivation of voltage-dependent Na^+^ channels. Thus, to examine the effects of ADP on activity-dependent modulation of action potentials, we first examined whether steady-state depolarization affects the action potentials (Fig. 5*A, B*). The amplitude of the action potential was measured from the baseline before stimulation because it was difficult to detect the threshold of propagating action potentials because of their smooth rising phase. The small depolarization preceding AP (13.4 ± 0.8 mV from control) caused no significant change in the peak amplitude (to 97.7 ± 2.9% of control) or the half-width of action potentials (to 97.8 ± 2.3% of control; *n* = 14, *p* = 0.542 and 0.326). The latency to the onset of action potentials was shortened to 85.9 ± 5.7% of the control (*n* = 14, *p* = 0.00842).

We then examined how ADP affects the subsequent action potentials. For this purpose, we recorded consecutive action potentials in response to the paired-pulse stimuli at short intervals (Fig. 5*C*). The peak membrane potential was slightly reduced to 96.8 ± 1.1% of the control at a 10-ms interval (*n* = 8, *p* = 0.0173, Fig. 5*D*) but was not significantly changed at longer intervals (to 99.7 ± 0.5%, 100.0 ± 0.2%, and 99.8 ± 0.2% of the control at 20-, 50-, or 100-ms intervals, respectively; *p* = 1, 0.889, and 0.674 for 20-, 50-, and 100-ms, *n* = 8, Fig. 5*D*). The peak reduction at the 10-ms interval was possibly due to incomplete recovery from the inactivation of Na^+^ channels (Engel and Jonas, 2005). We also examined the absolute value of the amplitude of action potentials. The amplitude of the second action potential was significantly reduced at 10-, 20-, and 50-ms intervals (to 86.8 ± 2.3%, 91.0 ± 1.4%, and 97.2 ± 0.6% of the control, *p* = 0.00195, 0.000479, and 0.00352, respectively), and it almost recovered to the basal level at a 100-ms interval (to 99.5 ± 0.5%, *p* = 0.287979), as shown in Fig. 5*D*. These time courses of depression of the second action potentials were similar to those of ADP, which lasted for several tens of milliseconds.

We recently reported that axonal spikes recorded by loose-patch-clamp recording were markedly depressed in response to paired-pulse stimuli (Ohura and Kamiya, 2018). As the derivative of action potentials was found to be proportional to the extracellularly recorded axonal spikes by the loose-patch configuration (Meeks et al., 2005; Ohura and Kamiya, 2018), we calculated the first derivative of the action potential by whole-cell recording (Fig. 5*E*). The second amplitude of the derivative of action potentials (Fig. 5*F*) was slightly lower than the first (88.4 ± 4.1%, 92.6 ± 1.5%, 96.4 ± 0.9%, and 97.8 ± 0.5% of control for 10-, 20-, 50-, and 100-ms intervals, respectively). The differences were significant (*p* = 0.00781, 0.00781, 0.00781, and 0.00781, for 10-, 20-, 50-, and 100-ms intervals, *n* = 8). The time course was consistent with that of axonal spikes recorded by loose-patch-clamp recording (Ohura and Kamiya, 2018).

We also examined the changes in duration of action potentials by paired stimuli. Repolarization was slightly slowed and the half-width of the action potentials was prolonged at short intervals (113 ± 5, 110 ± 2, and 104 ± 1% of the control at 10-, 20-, and 50-ms intervals, *p* = 0.00781, 0.00781, and 0.00781, respectively), but the difference was not significant at a 100-ms interval (102 ± 1% of control, *p* = 0.148), as shown in Fig. 5*G, H*. These findings were consistent with the previous study demonstrating activity-dependent broadening of action potentials by cumulated inactivation of potassium channels (Geiger and Jonas, 2000).

### Facilitation of presynaptic Ca^2+^ current by ADP

**Figure 6.**
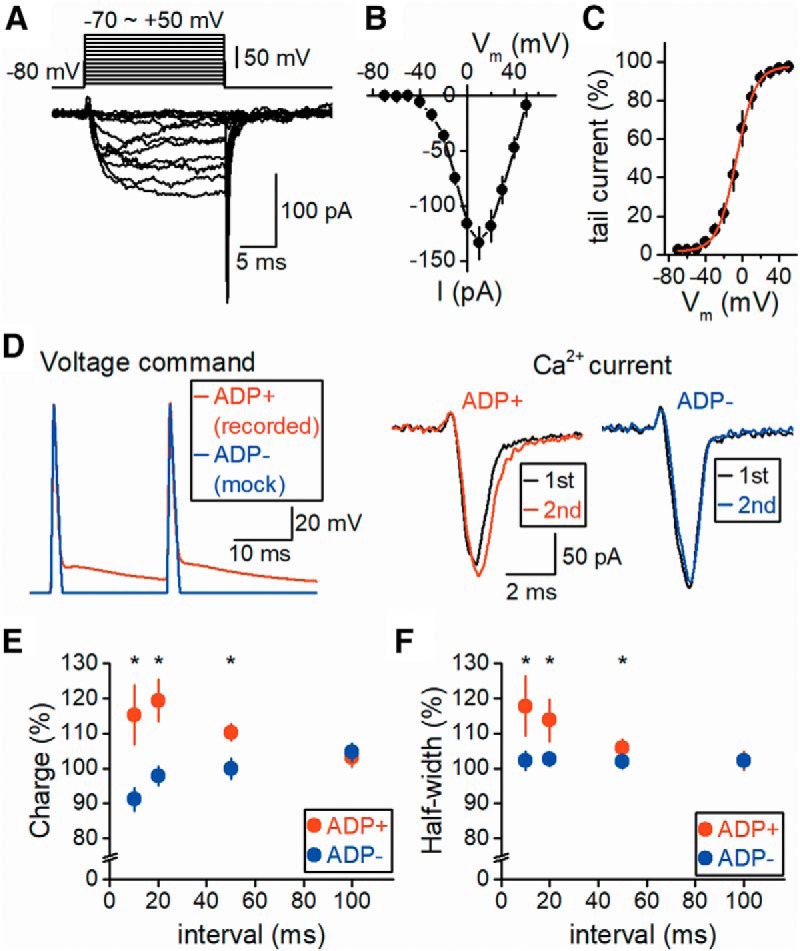
Facilitation of presynaptic Ca^2+^ influx by the axonal ADP. ***A***, Ca^2+^ currents in MFBs evoked by voltage steps from –80 mV to –70∼50 mV for 20 ms. ***B***, The averaged I-V relationships of presynaptic Ca^2+^ currents at MFBs. Current amplitudes were measured at the end of the voltage steps. ***C***, Voltage-dependence of activation of Ca^2+^ currents as shown by the amplitudes of tail currents. Data were normalized to the maximal value in each experiment and were fitted using a Boltzmann function (*n* = 7). ***D***, Voltage-clamp recording of Ca^2+^ currents using voltage commands mimicking the action potential waveforms elicited by paired-pulse stimuli (red). For comparison, mock action potential–like voltage commands mimicking broadening of action potentials by 10% in accordance with data in Fig. 5*H*, but lacking ADP, were applied (blue). Superimposed Ca^2+^ currents evoked by the first and second voltage commands are shown in the right panels. ***E***, ***F***, Summary data for the paired-pulse ratio of charge transfer (***E***) and half-width (***F***) of Ca^2+^ currents (*n* = 7, *, *p* < 0.05).

Lastly, we examined whether ADP modifies the presynaptic Ca^2+^ current, which directly controls transmitter release and short-term plasticity. Pharmacologically isolated Ca^2+^ currents (Fig. 6*A*) exhibited a typical time course of slow activation followed by fast tail currents, as in the previous study (Bischofberger et al., 2002). The current−voltage (I-V) relationship was also similar to that reported previously. Ca^2+^ currents were activated from approximately –40 mV and reached a maximum amplitude of 134 ± 15 pA at 10 mV (*n* = 7), as shown in Fig. 6*B*. The voltage-dependence of the activation of Ca^2+^ current was also estimated by measuring tail currents and plotted against the voltage (Fig. 6*C*). The steady-state activation data were fitted by a Boltzmann function with a slope factor of 10.4 mV and midpoint potential of –7.0 mV. These profiles of presynaptic Ca^2+^ currents in the MFBs were consistent with those reported in previous studies (Bischofberger et al., 2002; Li et al., 2007).

To explore the influence of ADP on the presynaptic Ca^2+^ influx, voltage-clamp recordings of Ca^2+^ currents were made using voltage commands mimicking the action potential waveforms recorded by paired-pulse stimuli shown in Fig. 5*C*. The charge transfer of Ca^2+^ currents was measured as the integrated area under the curve. The half-width of Ca^2+^ currents was also measured as the duration between the time points of the half and peak heights. The Ca^2+^ currents elicited by the second action potential voltage-commands with ADP at 10 ms were slightly facilitated compared with those elicited by the first action potential commands at 115.2 ± 8.5% of the control, and the half-width increased to 117.8 ± 7.7% (*n* = 7). These effects depended on the intervals of paired stimuli and were abolished at a 100-ms interval (to 103.0 ± 2.7% for the charge and to 102.1 ± 0.6% for the half-width). We considered this Ca^2+^ current facilitation by paired-pulse action potentials to be due to the broadening of action potentials, as shown in Fig. 5*G*, or facilitation of Ca^2+^ channels by prolonged depolarization during ADP. To investigate these possibilities, “mock” action potential-like voltage commands mimicking broadening of action potential but lacking ADP were applied to measure Ca^2+^ currents for comparison (Fig. 6*D*). In these recordings, Ca^2+^ currents were not facilitated (to 91.2 ± 3.3% and 104.6 ± 2.6% for 10- and 100-ms intervals, respectively, *n* = 7). At a 10-ms interval, the difference in the facilitation of Ca^2+^ current between with and without ADP was significant (*p* = 0.0156). The half-width of the Ca^2+^ current was unaffected by mock action potentials with ADP (to 102.2 ± 2.8% and 102.1 ± 2.0% for 10- and 100-ms intervals, respectively, *n* = 7). These results suggest that slight broadening of action potentials minimally affects Ca^2+^ entry, but ADP itself facilitates it, possibly by a mechanism similar to those recently reported at presynaptic terminals of cerebellar Purkinje cells (Zorrilla de San Martin et al., 2017). Although the underlying mechanisms remain to be elucidated, ADP following action potentials modulates Ca^2+^ entry into the presynaptic terminals by the second action potential, and may affect the short-term facilitation of transmitter release.

## Discussion

In this study, we examined the mechanisms underlying the prominent ADP following axonal action potentials using direct whole-cell recordings from hippocampal mossy fiber terminals in mice. We found that the axonal ADP was generated predominantly by slow capacitive discharge of the axonal membrane, reflecting passive propagation of action potentials. Slow activating Na^+^ channels also partly increase the capacitive component of the axonal ADP. On the other hand, voltage-dependent K^+^ channels curtail the initial phase of ADP by accelerating repolarization. Furthermore, we also examined the functional consequences of axonal ADP in the regulation of presynaptic functions. We found that prolonged subthreshold depolarization of axonal ADP facilitates the presynaptic Ca^2+^ current, thereby possibly modulating short-term synaptic plasticity.

### Components of axonal ADP

Action potentials recorded from the soma were often followed by ADP lasting for several tens of milliseconds, and were considered to regulate the fidelity of successive neuron firing during repetitive stimuli (Schwartzkroin, 1975; Storm, 1987). In addition, ADP is also known to be accompanied by action potentials propagating along axons. Thus far, axonal ADP was observed in several preparations allowing for direct recording from the axon and terminals such as in lizard motor axons (Barrett and Barrett, 1982), calyx of Held axon terminals (Borst et al., 1995), and hippocampal mossy fiber terminals (Geiger and Jonas, 2000). Therefore, the mechanisms of axonal ADP have been studied thoroughly, but remain somewhat controversial (Borst et al., 1995; Kim et al., 2010; Sierksma and Borst, 2017).

Regarding the mechanisms of axonal ADP, it has been widely accepted that the slow capacitive discharge of propagating action potentials contributes substantially to ADP (Barrett and Barrett, 1982), as expected from the cable properties of axons. As focal application of TTX minimally affected ADP at calyx of Held axon terminals (Borst et al., 1995), axonal ADP may be caused by, at least in part, a passive component due to capacitive discharge. In accordance with this notion, we found that the time course of slow repolarization of capacitive components, as measured in TTX and 4-AP conditions, was almost comparable to that of ADP.

On the other hand, ADP also demonstrated clear voltage-dependence, as shown in Fig. 2, which is different from the passive nature of the capacitive component. Therefore, involvement of voltage-dependent conductance in the generation of ADP is reasonable. In support of this notion, veratridine, which has complex effects on Na^+^ channels but exerts its facilitating action mainly by inhibiting inactivation (Ulbricht, 1998), robustly enhanced ADP, and it sometimes became overlaid with multiple spikes. In contrast, blocking Na^+^ channels with TTX partly reduced ADP. These results were consistent with the contribution of some slowly activating voltage-dependent Na^+^ channels to the generation of ADP, as reported in the calyx of Held axon terminals (Kim et al., 2010).

Previous studies demonstrated that slowly activating sodium channels, such as persistent-type I_NaP_ (Yue et al., 2005; D’Ascenzo et al., 2009; Kole, 2011; Ghitani et al., 2016) or resurgent-type I_NaR_ (Raman and Bean, 1997), different from transient-type I_NaT_ responsible for generation of action potentials, are involved in ADP. We previously reported that ADP in the granule cell soma in the dentate gyrus, which was reported to highly express I_NaR_ (Castelli et al., 2007), was markedly enhanced by veratridine (Ohura and Kamiya, 2018). This implies that ADP in hippocampal mossy fiber terminals may also be mediated by resurgent-type I_NaR_. Direct testing for the presence of I_NaR_ in future studies will help to reveal the contribution of specific Na^+^ channels to ADP at hippocampal mossy fibers.

We also found that voltage-dependent K^+^ channels sensitive to 4-AP are involved in the shaping of ADP. It was previously demonstrated that repolarization of action potentials at the MFBs is regulated by K_V_1 (Geiger and Jonas, 2000) and K_V_3 (Alle et al., 2011) voltage-dependent K^+^ channels. K^+^ currents elicited by action potential waveform commands are sensitive to 4-AP and were fully suppressed at 1 mM (Alle et al., 2011). We confirmed that 4-AP at 2 mm substantially delayed the repolarization of mock action potentials elicited in the presence of TTX, and the time course of residual components was voltage-independent, as shown in Fig. 4*E*. These results suggest that voltage-dependent K^+^ channels are also involved in the early phase of ADP to shorten the prolonged depolarization. This is consistent with the finding that ADP at the calyx of Held axon terminals is also negatively controlled by Kv1.1/1.2-type K^+^ channels to prevent multiple spikes during ADP (Dodson et al., 2003). The contribution of K_V_7 should be examined in a future study, as it was found to alter the somatic ADP in pyramidal cells (Yue and Yaari, 2004; Gu et al., 2005; Brown and Randall, 2009) and is present on hippocampal mossy fibers (Martinello et al., 2015).

Taken together, we consider ADP at hippocampal mossy fibers to consist of passive and active components. Passive capacitive discharge dominates and determines the time course of ADP. As active components, possible slow activating voltage-dependent Na^+^ channels sensitive to veratridine and TTX partly amplify the slow capacitive component. Voltage-dependent K^+^ channels sensitive to 4-AP also participate in shaping the early phase of ADP at hippocampal mossy fibers.

This interpretation may clarify the voltage-dependence of axonal ADP (Fig. 2). In several previous studies, the voltage-dependence of axonal ADP on changes in membrane potentials was reported, and “apparent” reversal potentials also varied between different preparations (Begum et al., 2016; Sierksma and Borst, 2017). The differential contribution of capacitive mechanism, as well as Na^+^ and K^+^ channel–dependent mechanisms, may explain the complex voltage-dependence of axonal ADP. Our study clarified that sodium channel–dependent and –independent mechanisms underlie ADP at hippocampal mossy fibers.

### Role of ADP in activity-dependent modulation of axonal action potentials

The functional consequence of ADP on subsequent axonal action potentials was examined thoroughly in previous studies. It was reported that ADP plays pivotal roles in ensuring reliable firing on multiple stimuli (Borst et al., 1995; Kim et al., 2010), as well as in modulating firing patterns (Bean, 2007; Sierksma and Borst, 2017). In this study, we examined activity-dependent modulation of the action potential waveform by ADP of preceding action potentials. ADP exhibited minimal effects on the peak of successive action potentials, except at very short intervals of 10 ms, when Na^+^ channels are not fully recovered from inactivation by the preceding action potentials (Brody and Yue, 2000; He et al., 2002; Engel and Jonas, 2005). However, peaks of the calculated first derivatives of action potentials, which are considered to reflect the maximal rate of the rising phase of action potentials, demonstrated paired-pulse depression for up to 100 ms. This is consistent with our previous observation that axonal spikes recorded extracellularly using loose-patch clamp recording had paired-pulse depression following a similar time course (Ohura and Kamiya, 2018), because extracellularly recorded axonal spikes are theoretically proportional to the first derivative of action potentials (Meeks et al., 2005).

Furthermore, activity-dependent broadening of action potentials (Geiger and Jonas, 2000) was detectable even in paired-pulse protocols in this study. As suggested, accumulated inactivation of K_V_1-type voltage-dependent K^+^ channels may cause activity-dependent broadening of action potentials at hippocampal mossy fibers. A similar mechanism may underlie ADP, although the magnitude of broadening is much smaller.

### Role of ADP in facilitation of the presynaptic Ca^2+^ current

The shape of the presynaptic action potential determines the calcium influx to the presynaptic terminals, and thereby modulates transmitter release from the presynaptic terminals. Broadening of action potentials during repetitive stimuli was reported to enhance the calcium influx into presynaptic terminals (Geiger and Jonas, 2000; Bischofberger et al., 2002). Even in the paired-stimuli conditions, mild broadening of action potentials was observed, but it minimally affected the Ca^2+^ current elicited by mock action potentials without ADP, as shown in Fig. 6*D*. Although it is unclear why the Ca^2+^ current was not facilitated by paired-pulse, the positive effects of spike broadening may be counteracted or cancelled by residual inactivation of Ca^2+^ channels at short intervals. Slight changes in action potential rise times may also contribute to this apparent discrepancy.

On the other hand, Ca^2+^ currents elicited by voltage-clamp commands of the recorded action potentials with ADP were slightly but significantly facilitated. Recently, direct recording from Purkinje cell axon terminals revealed that prior subthreshold depolarization facilitated Ca^2+^ currents (Zorrilla de San Martin et al., 2017). Although the detailed biophysical mechanism awaits future investigation, a similar mechanism may underlie the paired-pulse facilitation of Ca^2+^ currents observed in this study.

### Functional implications

Axonal ADP following action potentials is a fundamental and important upstream process of transmitter release, which accordingly affects short-term synaptic plasticity. In this study, we found that the axonal ADP may facilitate presynaptic Ca^2+^ currents elicited by action potentials. As it has been demonstrated that somatic depolarization also affects transmitter release from MFBs in a Ca^2+^-independent manner (Scott et al., 2008), Ca^2+^ current facilitation as well as Ca^2+^-independent facilitation of transmitter release may be involved in the activity-dependent short-term plasticity at synapses. Simultaneous recording from both presynaptic MFBs and postsynaptic pyramidal cells (Vyleta and Jonas, 2014; Midorikawa and Sakaba, 2017) to quantitatively correlate presynaptic Ca^2+^-signaling with postsynaptic EPSPs will clarify the detailed functions of axonal ADP for the short-term plasticity at hippocampal mossy fiber synapses.

## Synthesis

Reviewing Editor: Pablo Castillo, Albert Einstein College of Medicine Decisions are customarily a result of the Reviewing Editor and the peer reviewers coming together and discussing their recommendations until a consensus is reached. When revisions are invited, a fact-based synthesis statement explaining their decision and outlining what is needed to prepare a revision will be listed below. The following reviewer(s) agreed to reveal their identity: Peter Jonas, J. Borst. Note: If this manuscript was transferred from JNeurosci and a decision was made to accept the manuscript without peer review, a brief statement to this effect will instead be what is listed below.

The reviewers agree that your revised manuscript has improved significantly. However, as indicated below, they also understand (and I concur) the text must be thoroughly edited by a native English speaker. In addition, you must address all specific points raised by the reviewers --please, cite each original comment.

-----------------------------------------------------------------

Reviewer #1

The paper by Ohura and Kamiya has been substantially improved. The language is not great, but acceptable. I have only a couple of relatively minor suggestions.

Line 183: ‘were perfused’ should read ‘was perfused’.

Line 194: Shouldn't a small depolarizing holding current be necessary when the initial resting membrane potential was -85 mV?

Line 211: How do the authors know that this is due to spiking of the neighboring axons or boutons?

Line 296: Li et al., 2007, should be cited here.

Line 654: Slight changes in action potential rise time or peak amplitude may also contribute to this apparent discrepancy. This might be mentioned.

The authors should provide a visual abstract.

Reviewer #2

Ohura and Komiya have revised their paper about the ionic and electrical mechanisms underlying the afterdepolarization (ADP) in hippocampal mossy fiber boutons (MFBs) and its impact on the Ca current, and submitted it to eNeuro. They have added new experiments in which they addressed the dependence of the ADP on the presence of extracellular Ca, and improved the analysis and in some cases the wording of their paper.

Major points:

In my original review I wrote that “Most sentences contain at least one grammatical error, and often it is hard to deduce what the authors are trying to say.” The authors responded by correcting “all the point according to the both reviewers' suggestions”. They also “edited throughout the manuscript”, sometimes introducing new errors (e.g. last sentence of the Abstract). As a result, many sentences still contain at least one grammatical error, and it is often still hard to deduce what the authors are trying to say.

Judging the rebuttal was not always easy, since the authors did not cite the original comments, making it hard to find out which comment they are addressing. Moreover, the yellow highlighting in their revised manuscript does not always mark changed text, and I had to do a ‘compare documents’ to find out what was changed. Despite these difficulties, most points were satisfactorily addressed in their rebuttal and revised manuscript. The new experiments are a useful addition. The Ca tail currents now look much better. The statistics associated with Figure 2C now seem to be in order.

I believe that in the revised manuscript, the ‘N’ now always refers to the number of independent observations. I am still unsure why the authors exclusively use non-parametric tests.

## Author Response

Responses to the reviewer #1

1. Line 183: “were perfused” should read “was perfused”.

The reviewer kindly suggested correction of grammar. We edited as suggested.

(Line 179 in the revised manuscript).

2. Line 194: Shouldn't a small depolarizing holding current be necessary when the

initial resting membrane potential was -85 mV?

The reviewer kindly pointed out the unclear description. We injected either

hyperpolarizing or depolarizing to set the membrane potential to -80 mV. Therefore,

we removed the word “hyperpolarizing”. (Line 191-192 in the revised manuscript)

3. Line 211: How do the authors know that this is due to spiking of the neighboring

axons or boutons?

The reviewer suggested to explain why we considered the very slow tail currents

observed in some recordings are due to spiking of unclamped neighboring axons or

boutons. These slow tail currents appeared in an all-or-none manner, and therefore we

considered that they reflect spiking of neighboring axons or boutons. (Line 207-208 in

the revised manuscript)

4. Line 296: Li et al., 2007, should be cited here.

The reviewer suggested to cite Li et al. 2007 as a reference for presence of R-type

Ca channels at hippocampal mossy fiber terminals. We cited the paper. (Line 292 in

the revised manuscript)

2

5. Line 654: Slight changes in action potential rise time or peak amplitude may also

contribute to this apparent discrepancy. This might be mentioned.

According to the reviewer's suggestion, we mentioned about the possibility. (Line

647-648 in the revised manuscript)

6. The authors should provide a visual abstract.

A visual abstract was provided.

Responses to the reviewer #2

1. In my original review I wrote that “Most sentences contain at least one

grammatical error, and often it is hard to deduce what the authors are trying to

say.” The authors responded by correcting “all the point according to the both

reviewers' suggestions”. They also “edited throughout the manuscript”, sometimes

introducing new errors (e.g. last sentence of the Abstract). As a result, many

sentences still contain at least one grammatical error, and it is often still hard to

deduce what the authors are trying to say.

According to the reviewer's comment, our manuscript was corrected by a native

English speaker.

2. Judging the rebuttal was not always easy, since the authors did not cite the

original comments, making it hard to find out which comment they are

addressing. Moreover, the yellow highlighting in their revised manuscript does not

3

always mark changed text, and I had to do a ‘compare documents’ to find out what

was changed. Despite these difficulties, most points were satisfactorily addressed

in their rebuttal and revised manuscript. The new experiments are a useful

addition. The Ca tail currents now look much better. The statistics associated with

Figure 2C now seem to be in order.

In this rebuttal, we cited each original comment of the reviewers. We highlighted

the changes in the revised manuscript, as written in the response to the reviewer #1,

except for the grammatical corrections suggested by the English editing service.

3. I believe that in the revised manuscript, the ‘N’ now always refers to the number

of independent observations. I am still unsure why the authors exclusively use

non-parametric tests.

The reviewer asked why we used non-parametric tests for statistical comparison of

two groups. Since it was hard to obtain enough sample numbers to pass a normality

test in whole-bouton recording experiments, we adopted non-parametric test in this

study.
